# IL-17C contributes to NTHi-induced inflammation and lung damage in experimental COPD and is present in sputum during acute exacerbations

**DOI:** 10.1371/journal.pone.0243484

**Published:** 2021-01-07

**Authors:** Giovanna Vella, Felix Ritzmann, Lisa Wolf, Andreas Kamyschnikov, Hannah Stodden, Christian Herr, Hortense Slevogt, Robert Bals, Christoph Beisswenger

**Affiliations:** 1 Department of Internal Medicine V – Pulmonology, Allergology and Respiratory Critical Care Medicine, Saarland University, Homburg, Germany; 2 Septomics Research Center, Jena University Hospital, Jena, Germany; Institute of Lung Biology and Disease (iLBD), Helmholtz Zentrum München, GERMANY

## Abstract

Neutrophilic inflammation results in loss of lung function in chronic obstructive pulmonary disease (COPD). Gram-negative bacteria, such as nontypeable *Haemophilus influenzae* (NTHi), trigger acute exacerbations of COPD (AECOPD) and contribute to chronic lung inflammation. The pro-inflammatory cytokine interleukin-17C (IL-17C) is expressed by airway epithelial cells and regulates neutrophilic chemotaxis. Here, we explored the function of IL-17C in NTHi- and cigarette smoke (CS)-induced models of COPD. Neutrophilic inflammation and tissue destruction were decreased in lungs of IL-17C-deficient mice (*Il-17c*^*-/-*^) chronically exposed to NTHi. Numbers of pulmonary neutrophils were decreased in *Il-17c*^*-/-*^ mice after acute exposure to the combination of NTHi and CS. However, *Il-17c*^*-/-*^ mice were not protected from CS-induced lung inflammation. In a preliminary patient study, we show that IL-17C is present in sputum samples obtained during AECOPD and associates with disease severity. Concentrations of IL-17C were significantly increased during advanced COPD (GOLD III/IV) compared to moderate COPD (GOLD I/II). Concentrations of IL-17A and IL-17E did not associate with disease severity. Our data suggest that IL-17C promotes harmful pulmonary inflammation triggered by bacteria in COPD.

## Introduction

Cigarette smoke (CS) and exposure to indoor air pollution are the major risk factors for the development of chronic obstructive pulmonary disease (COPD) which is expected to become the fourth leading cause of death worldwide by 2030. COPD is characterized by chronic lung inflammation which associates with increased numbers of pulmonary neutrophils, macrophages, and T lymphocytes [1, 2]. Lungs of COPD patients are frequently infected with Gram-negative bacteria, such as nontypeable *Haemophilus influenzae* (NTHi), which amplify chronic lung inflammation and therefore contribute to tissue destruction and loss of lung function. In addition, lung infections are associated with acute exacerbation of COPD (AECOPD) [1, 2].

The pro-inflammatory cytokine IL-17C belongs to the IL-17 cytokine family and it is primary expressed by epithelial cells [3–7]. IL-17C shares the IL-17 receptor IL-17RA with IL-17A and regulates inflammation in an autocrine manner through the induction of cytokines, chemokines, and antimicrobial peptides [3–5, 8–10]. The expression of IL-17C in airway epithelial cells is induced by microbial factors and inflammatory cytokines (e.g. IL-17A, TNF-α) [5, 6, 8, 11].

There is evidence that IL-17C plays a role in COPD. A recent study found that IL-17C is strongly expressed in pathogenic stem cells in lungs of COPD patients [12]. Tissue culture studies showed that rhinovirus and bacteria synergistically induce the expression of IL-17C in bronchial epithelial cells and that epithelial cells obtained from COPD patients secret increased amounts of IL-17C compared to cells from non-COPD patients [11]. In addition, knockdown of IL-17C in cultured bronchial epithelial cells reduced the induction of chemokines in response to infection and neutrophil chemotaxis [11]. We have shown that IL-17C mediates the recruitment of neutrophils during experimental *Streptococcus pneumoniae* and *Pseudomonas aeruginosa* pneumonia and inflammation induced-recruitment of neutrophils into the microenvironment of lung tumors [6, 13, 14].

The purpose of this study was to further characterize the function of IL-17C in COPD. We show that IL-17C mediates the NTHi-induced expression of neutrophilic cytokines, the recruitment of neutrophils, and lung damage in models of experimental COPD. We demonstrate that IL-17C is present in sputum of COPD patients during AECOPD and provide evidence that concentrations of IL-17C associate with disease severity.

## Materials and methods

### Patient study

The protocol was approved by the ethics committee (Institutional Review Board) of the Landesärztekammer des Saarlandes and informed written consent was obtained from all patients. No minors were included into the study. Spontaneous sputum was collected from patients during AECOPD. AECOPD was defined as a condition with acute increase of respiratory symptoms (increased sputum production, cough, dyspnea) and the exclusion of differential diagnoses. Clinical data on pulmonary function were determined during a stable phase of the disease. Sputum plugs were collected and weighted. The sputum plugs were incubated with 2 ml sputulysin (0.1% DTT in RNase free water) per gram sputum plugs for 15 minutes at room temperature (RT). PBS was added at a ratio of 1:1 and the samples were vortexed for 15 seconds. The samples were centrifuged for 10 min (800 x g, RT) and the supernatant was frozen at -80°C. Concentrations of IL-17A, IL-17C, IL-17E, IL-8, and CXCL5 were analyzed using magnetic luminex assay (R&D Systems, Minneapolis, MN, USA) using MAGPIX (Luminex corporation, Austin, USA).

### Mouse experiments

All animal experiments were approved by the Landesamt für Soziales, Gesundheit und Verbraucherschutz of the State of Saarland in agreement with the national guidelines for animal treatment. IL-17C-deficient (*Il-17c*^*-/-*^) C57BL/6 mice were obtained from Mutant Mouse Resource and Research Center (MMRRC, USA). Power analysis based on effect size estimates was performed to determine sample size estimation prior to the experiments. For experiments 8 to 10 weeks old female wildtype (WT) and *Il-17c*^*-/-*^ mice were exposed to NTHi as described before [13]. Briefly, the mice were exposed to sonicated and heat-inactivated clinical isolate of NTHi (2.5 mg/ml protein in PBS) for 40 minutes per day in a plexi glass box connected to a Pari MASTER^®^ nebulizer (Pari GmbH, Starnberg, Germany). In the chronic exposure model, mice were exposed three times per week to NTHi at days 1, 3, and 5 within the first 4 weeks and once a week at day 1 in the following 8 weeks. In the acute and chronic exposure model, the mice were exposed to smoke for two periods of 50 minutes with a resting phase of 2 hours between the smoke exposures (3R4F; College of Agriculture, Reference Cigarette Program, University of Kentucky, Lexington, KY) in a TE-10 (Teague Enterprises) smoking chamber (Teague Enterprises, Woodland, CA) at 5 days per week. The CO concentrations were between 200 and 350 ppm and the CS concentration was up to 120 mg/m^3^ total suspended particles during the smoking period. The mice were exposed to NTHi at days 1, 3, and 5, 1 to 2 hours after exposure to CS. Mice were analyzed 24 hours after the final exposure to NTHi.

### Histopathology

All histologic analysis were performed as described before [15]. In brief, lungs were fixed by instillation of a buffered 4% formaldehyde solution under a constant hydrostatic pressure (30 cm H_2_O for 15 minutes), kept in 4% formaldehyde solution for additional 24 hours without hydrostatic pressure, embedded in 1% agarose, cut into slices of exactly the same thickness (5 mm), and embedded in paraffin. Primary antibodies for CD3 (rabbit, 1/100, ab5690; Abcam, Cambridge, UK) and Ly6B (rat, 1/150, MCA771GA; Bio-Rad, Munich, Germany) were used for immunohistochemistry analysis blinded to the investigator. Corresponding HRP-conjugated secondary antibodies (anti-rabbit 414341F and anti-rat 414311F, Histofine Simple Stain, Nichirei Biosciences Inc. Japan) were used. Randomly selected fields were evaluated for positive cells using the Visiopharm Integrator System (Visiopharm, Hoersholm, Denmark) on an Olympus BX51 microscope. Paraffin sections were stained with hematoxylin-eosin (H&E), blinded to the investigator and the inflammatory score was calculated as described before [6, 16]. The mean chord length (MCL) was calculated blinded to the investigator using the Visiopharm Integrator System on an Olympus BX51 microscope equipped with an 8-position slide loader.

### Determination of immune cells, cytokine concentrations, and quantitative RT-PCR (qRT-PCR)

A bronchoalveolar lavage (BAL) was performed with 1 ml of PBS flushed three times into the lungs through the cannulated trachea [6]. Numbers of immune cells were determined in BAL fluids and cytospins were prepared for the differentiation of neutrophils, macrophages, and lymphocytes by light microscopy. In the acute exposure model, RNA was isolated from right lung lobes using the Trizol Reagent (Life Technologies, USA). The remaining left lung lobes were homogenated in 1 ml PBS. Cytokine concentrations were measured in BAL fluids and homogenated lungs using an enzyme-linked immunosorbent assay (ELISA, R&D Systems, Germany) as described before [6]. RNA was isolated using Trizol Reagent (Life Technologies, USA). Real-time PCR was performed as described before [14, 17–19]. PCR program with SensiMix SYBR Master Mix (Bioline, UK): initial denaturation step of 10 min at 95°C; 40 cycles of 15 sec at 95°C and 30 sec at 60°C. Primers were as follows: *GAPDH*, 5-ATG GTG AAG GTC GGT GTG-3 and 5-CAT TCT CGG CCT TGA CTG-3; *IL-17C*, 5-CTG GAA GCT GAC ACT CAC G-3 and 5-GGT AGC GGT TCT CAT CTG TG-3; *IL-6*, 5-AGT TGC CTT CTT GGG ACT GA-3 and 5-TCC ACG ATT TCC CAG AGA AC-3; *csf3*, 5-CAG CCC AGA TCA CCC AGA ATC-3 and 5-CTG CAG GGC CAT TAG CTT CAT-3; *KC*, 5-GCT GGG ATT CAC CTC AAG AA-3 and 5-AGG TGC CAT CAG AGC AGT CT-3. mRNA expression was normalized to GAPDH mRNA expression using the ΔΔCT method [20].

### Stimulation of primary alveolar epithelial cells

Primary murine alveolar epithelial cells were obtained as described before [15]. Only monolayers with a TEER (transepithelial electrical resistance) above 350 Ωcm^2^ were used for experiments. Sonicated and heat-inactivated clinical isolate of NTHi (0.25 mg/ml protein in PBS) or PBS were applied to the apical compartment for 24 hours. IL-17C mRNA expression was normalized to GAPDH mRNA expression using the ΔΔCT method [20].

### Statistical analysis

Correlation analysis was performed using nonparametric Spearman’s correlation test. Comparisons between groups were analyzed by appropriate nonparametric (Mann-Whitney) or parametric (one-way anova with bonferroni post hoc test, student t-test) tests using the software Prism (GraphPad Software, San Diego, CA). Results were considered statistically significant for p < 0.05.

## Results

### IL-17C regulates NTHi-induced chronic neutrophilic lung inflammation

**Fig 1:**
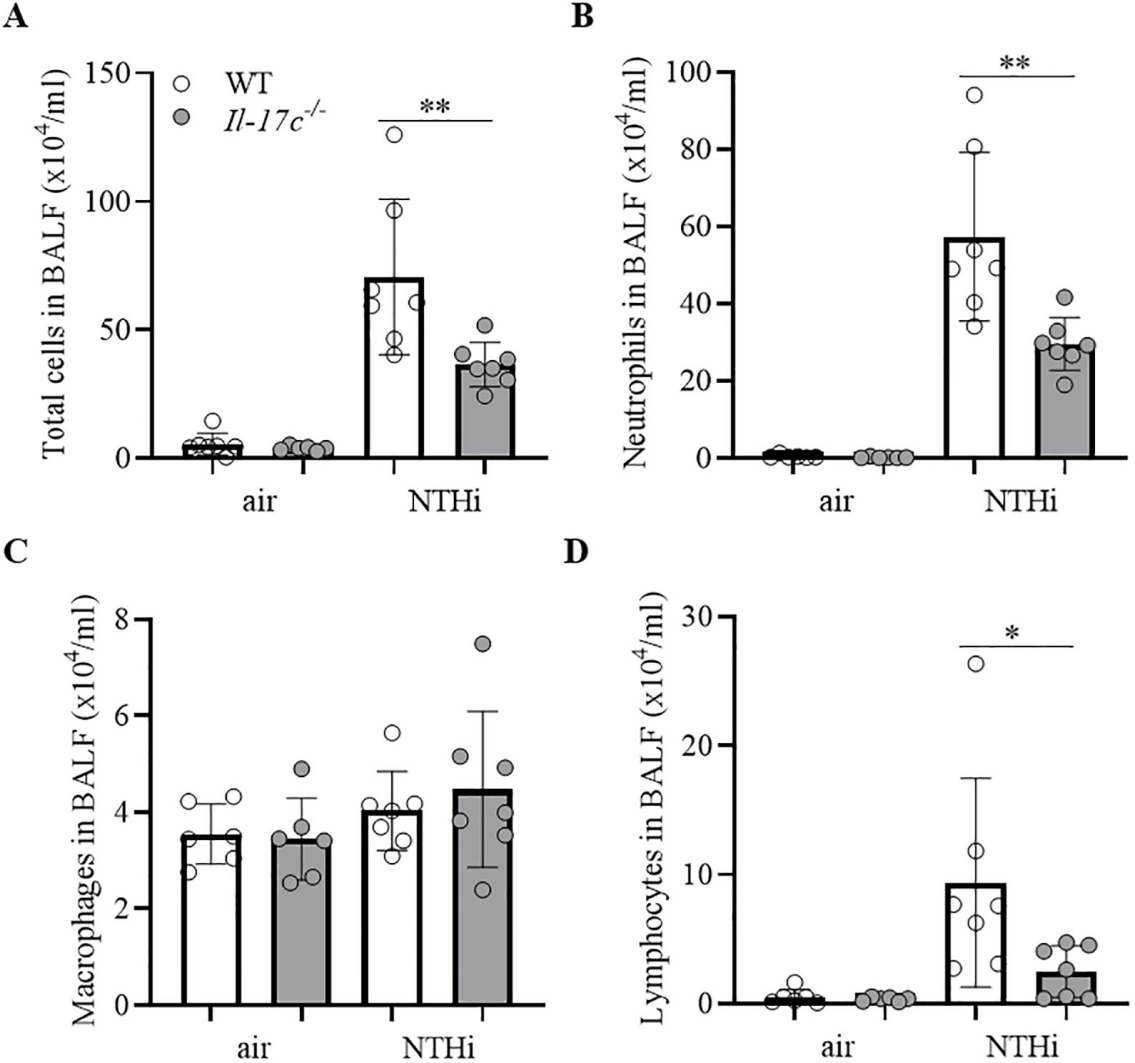
IL-17C mediates chronic neutrophilic inflammation. WT and *Il-17c*^*-/-*^ mice were exposed to heat-inactivated NTHi three times a week (day 1, 3, 5) for 4 weeks. Numbers of total immune cells (A), neutrophils (B), macrophages (C), and lymphocytes (D) were determined in BAL fluids 24 hours after the final exposure to NTHi (n = 6–7 per group). Data were compared by One-way ANOVA with Bonferroni post-test and are shown as the mean ± SD. *p < 0.05 and **p < 0.01.

**Fig 2:**
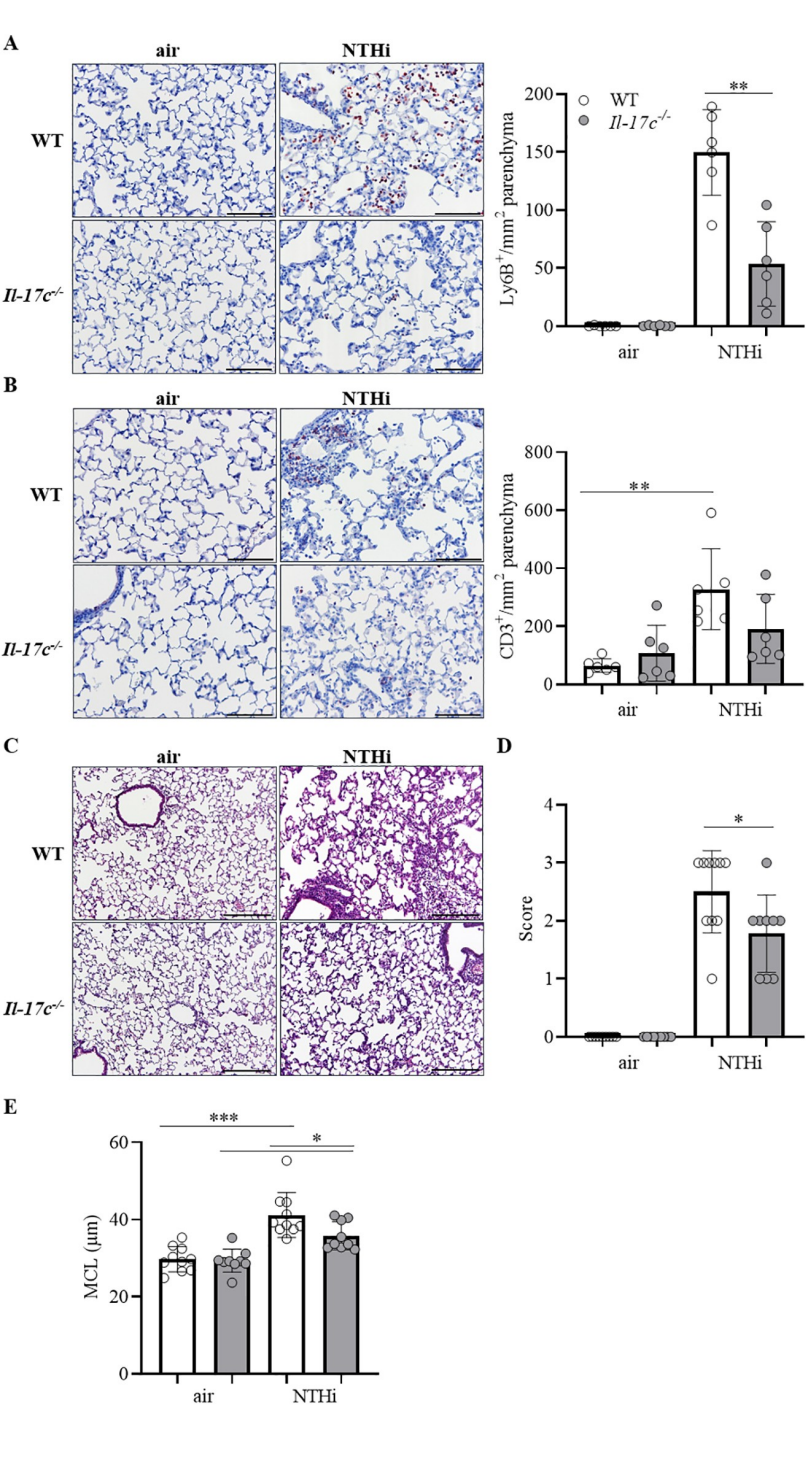
Reduced lung damage in mice deficient for IL-17C. WT and *Il-17c*^*-/-*^ mice were exposed to heat-inactivated NTHi three times a week (day 1, 3, 5) for 4 weeks. Mice were analyzed 24 hours after the final exposure to NTHi. Representative IHC of Ly6B (A) and CD3 (B) and quantification of the Ly6B^+^ and CD3^+^ cells in lung parenchyma (n = 6 mice per group, 6–8 fields per mouse, Scale bar: 50 μm). Representative lung histology (C, hematoxylin and eosin staining) and inflammatory score (D) (n = 9–10 per group, 2–3 lung sections per mouse, scale bar: 100 μm). Data were compared by unpaired Student’s t-test and are shown as the mean ± SD. *p < 0.05 and **p < 0.01. (E) The mean chord length (MCL, 27–50 fields per mouse, n = 9–10 per group) was determined by morphometric methods. Data were compared by One-way ANOVA with Bonferroni post-test and are shown as the mean ± SD. *p < 0.05 and ***p < 0.001.

Lungs of COPD patients are frequently infected with Gram-negative bacteria (e.g. NTHi) which trigger AECOPD and promote chronic lung inflammation (17). To study whether IL-17C contributes to inflammation-induced lung damage we exposed WT and *Il-17c*^*-/-*^ mice to heat-inactivated NTHi for 4 weeks. Our model of chronic NTHi-exposure is comparable with well-established LPS- and elastase-dependent models of experimental COPD and induces numerous characteristics of COPD, such as neutrophilic lung inflammation (Fig 1) and enlarged airspaces (Fig 2) [21–23]. Pulmonary inflammation was remarkably reduced in *Il-17c*^*-/*-^ mice. Numbers of total immune cells, neutrophils, and lymphocytes were significantly decreased in BAL fluids of *Il-17c*^*-/*-^ mice, whereas there were no significant differences in the numbers of macrophages after exposure to NTHi for four weeks (Fig 1). Similar results were obtained with WT and *Il-17c*^*-/*-^ mice exposed to NTHi for 12 weeks (S1 Fig, 10.6084/m9.figshare.13128278). We further analyzed Ly6B^+^ cells by immunohistochemistry (IHC). Ly6B is a marker for neutrophils and inflammatory monocytes [24]. There were nearly no Ly6B^+^ cells present in the parenchyma of WT and *Il-17c*^*-/*-^ control mice, whereas chronic inflammation resulted in enhanced numbers of segmented Ly6B^+^ cells in the parenchyma. Numbers of Ly6B^+^ cells were significantly decreased in the parenchyma of NTHi-exposed *Il-17c*^*-/*-^ mice compared to NTHi-exposed WT mice (Fig 2A). Chronic NTHi-induced inflammation resulted in significantly increased numbers of CD3^+^ cells in WT mice, but not in *Il-17c*^*-/*-^ mice (Fig 2B). Histological examination of the lungs showed that the inflammation-induced immune cell infiltration was attenuated in *Il-17c*^*-/*-^ mice (Fig 2C). The inflammatory score was significantly decreased in *Il-17c*^*-/*-^ mice (Fig 2D). Morphometry showed that the mean chord length (MCL) values in lungs from NTHi-exposed *Il-17c*^*-/*-^ mice were significantly decreased compared with NTHi-exposed WT mice indicating that deficiency for IL-17C partially protects loss of lung structure (Fig 2E).

**Fig 3:**
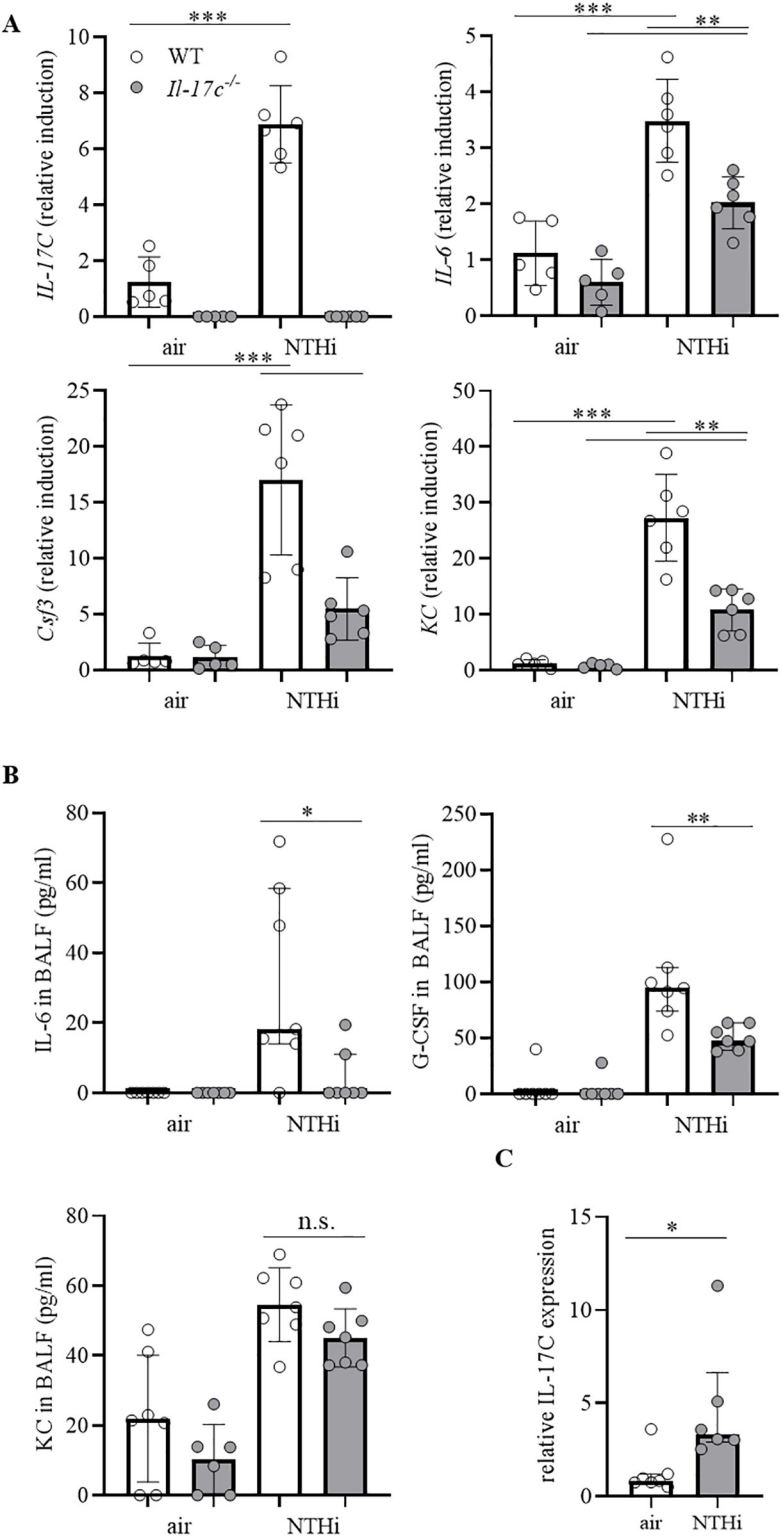
*Il-17c* deletion decreases the expression of inflammatory cytokines. WT and *Il-17c*^*-/*-^ mice were exposed to heat-inactivated NTHi three times a week (day 1, 3, 5) for 4 weeks. Mice were analyzed 24 hours after the final exposure to NTHi. (A) Relative expression of IL-17C, IL-6, G-CSF, and KC in lung tissue (n = 5–6 mice per group). Data were compared by One-way ANOVA with Bonferroni post-test and are shown as the mean ± SD. **p < 0.01, and ***p < 0.001. (B) Concentrations of IL-6, G-CSF, and KC in BAL fluids (n = 4–7 mice per group). (C) Polarized primary alveolar epithelial cells were stimulated with inactivated NTHi (0.25 mg/ml protein in PBS). The expression of IL-17C was measured by qRT-PCR after 24 hours. Data were compared by Mann-Whitney test and are shown as the median with interquartile range. p < 0.05 and **p < 0.01.

We analyzed the expression of inflammatory mediators in lungs of mice exposed to heat-inactivated NTHi for four weeks. The relative mRNA expression of IL-17C was increased in lungs of WT mice, whereas IL-17C mRNA was not detectable in *Il-17c*^*-/*-^ mice, verifying the expected results (Fig 3A). In addition, the relative mRNA expression of interleukin-6 (IL-6), granulocyte colony stimulating factor (G-CSF), and keratinocyte-derived chemokine (KC) was significantly decreased in lungs of NTHi-exposed *Il-17c*^*-/*-^ mice compared with NTHi-exposed WT mice (Fig 3A). Concentrations of IL-6 and G-CSF, but not KC were significantly decreased in BAL fluids of NTHi-exposed *Il-17c*^*-/*-^ mice (Fig 3B). We studied whether *NTHi* induces the expression of IL-17C in primary murine alveolar epithelial cells *in vitro*. Stimulation of polarized alveolar epithelial cells with NTHi resulted in a significantly increased expression of IL-17C (Fig 3C).

**Fig 4:**
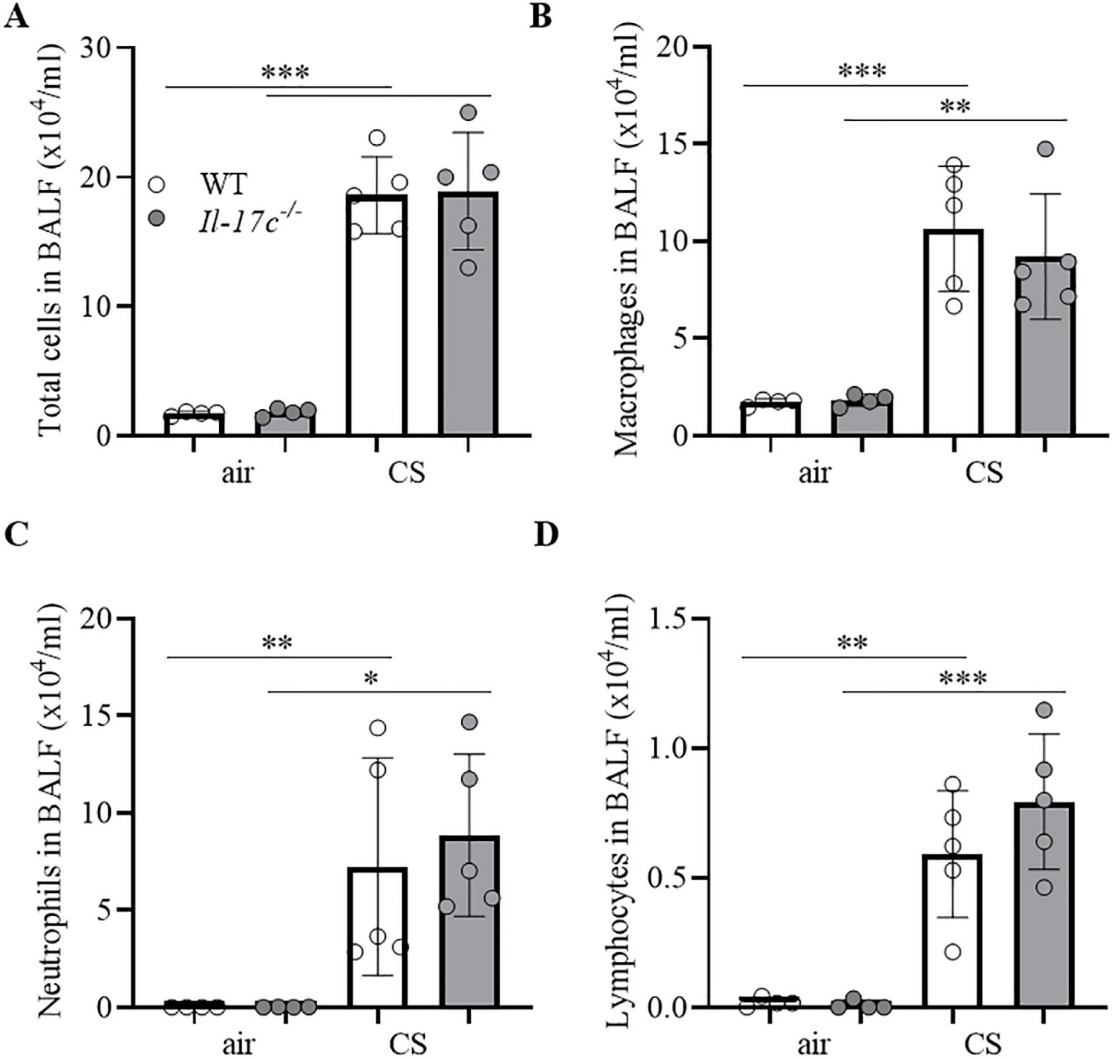
IL-17C does not contribute to CS-induced lung inflammation. WT and *Il-17c*^*-/-*^ mice were exposed to CS for 4 weeks five times a week (days 1–5). Numbers of total immune cells (A), macrophages (B), neutrophils (C), and lymphocytes (D) were determined in BAL 24 hours after the final exposure to CS (n = 4–5 mice per group). Data were compared by unpaired Student’s t-test and are shown as the mean ± SD. *p < 0.05, **p < 0.01, and ***p < 0.001.

We further examined whether IL-17C mediates CS-induced lung inflammation. Therefore, we exposed WT and *Il-17c*^*-/-*^ mice to CS for 4 weeks and analyzed the inflammatory cells in BAL fluids 24 hours after the final exposure to CS. Fig 4 shows that the deficiency for IL-17C did not affect the CS-induced increase in the numbers of inflammatory cells.

### IL-17C regulates acute NTHi-exacerbated lung inflammation

**Fig 5:**
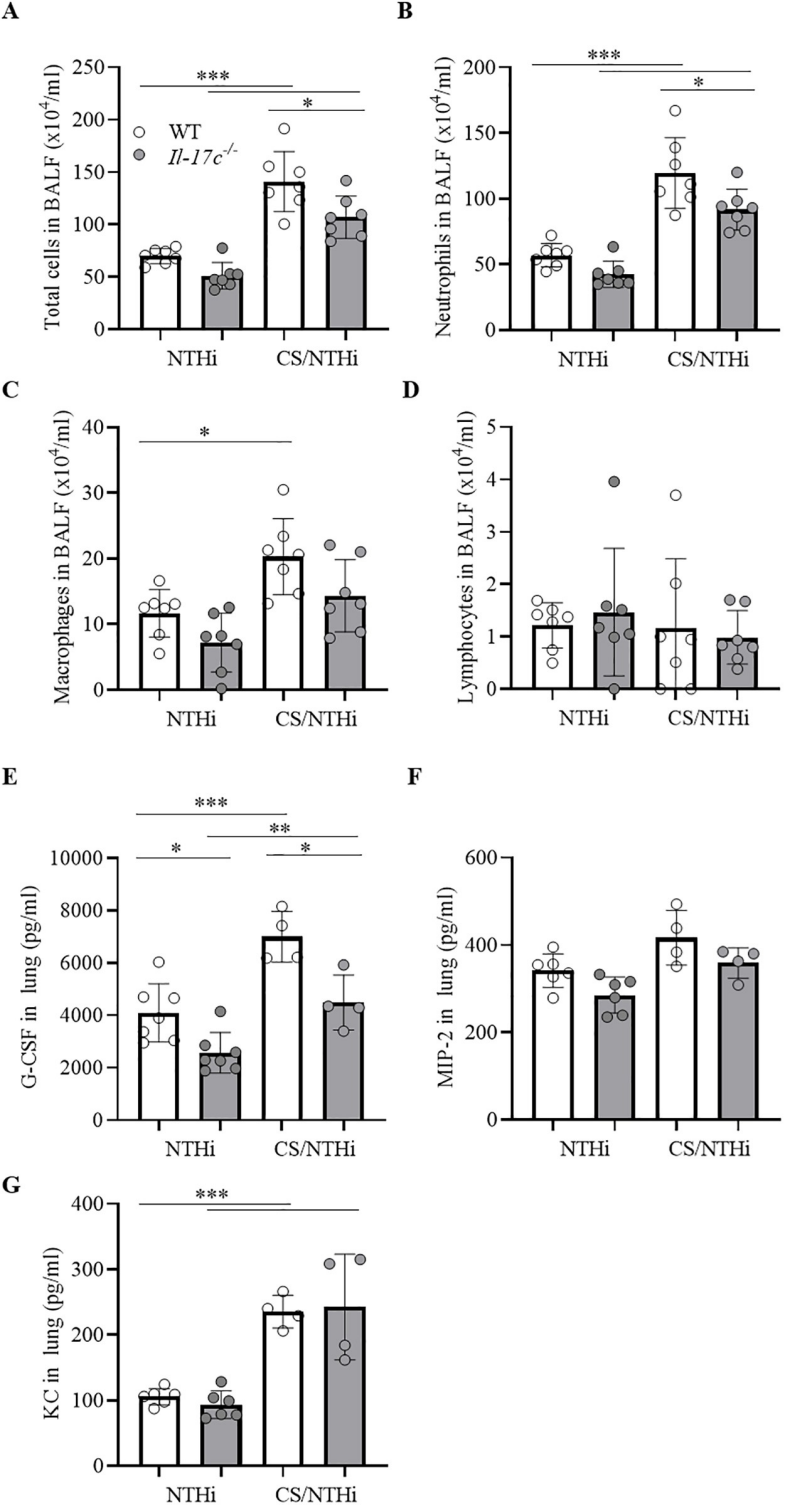
IL-17C contributes to acute COPD-like lung inflammation. WT and *Il-17c*^*-/-*^ mice were exposed to NTHi (day 1, 3, and 5) or the combination of NTHi (day 1, 3, 5) and CS (day 1 to 5). Numbers of total immune cells (A), neutrophils (B), macrophages (C), and lymphocytes (D) were determined in BAL fluids 24 hours after the final exposure to NTHi (n = 7 per group). Concentrations G-CSF (E), MIP-2 (F), and KC (G) were measured in lung homogenate (n = 4–7 mice per group). Data were compared by One-way ANOVA with Bonferroni post-test and are shown as the mean ± SD. *p < 0.05, **p < 0.01, and ***p < 0.001.

To investigate whether 17C promotes acute COPD-like lung inflammation we exposed mice to heat-inactivated NTHi (day 1, 3, and 5) and the combination of CS (day 1 to 5) and NTHi (day 1, 3, and 5) and analyzed the mice 24 hours after the final exposure to NTHi. Exposure to the combination of CS and NTHi resulted in significantly increased numbers of total cells in WT mice compared to NTHi-exposed mice (Fig 5A). Significantly increased numbers of BAL cells were also observed in CS/NTHi-exposed *Il-17c*^*-/-*^ mice. However, the total numbers of cells in BAL fluids of CS/NTHi-exposed *Il-17c*^*-/-*^ mice were significantly lower than the numbers of BAL cells found in CS/NTHi-exposed WT mice (Fig 5A). Differential cell counting revealed that the differences in the numbers of BAL cells between infected WT and *Il-17c*^*-/-*^ mice resulted from decreased recruitment of neutrophils and macrophages in *Il-17c*^*-/-*^ mice (Fig 5B and 5C). Numbers of neutrophils were significantly decreased in CS/NTHi-exposed *Il-17c*^*-/-*^ mice compared to the level in CS/NTHi-exposed WT mice. There were no significant differences in the numbers of lymphocytes between WT and *Il-17c*^*-/-*^ mice (Fig 5D). We further measured the concentrations of chemokines known to mediate the recruitment of neutrophils. Concentrations of G-CSF were significantly decreased in BAL fluids of NTHi-exposed and CS/NTHi-exposed *Il-17c*^*-/-*^ mice compared to WT mice (Fig 5E), whereas concentrations of the macrophage inflammatory protein 2 (MIP-2) and keratinocyte-derived chemokine (KC) were not significantly affected by the IL-17C deficiency at the time-point measured (Fig 5F and 5G).

### Sputum IL-17C levels are increased in advanced COPD

**Fig 6:**
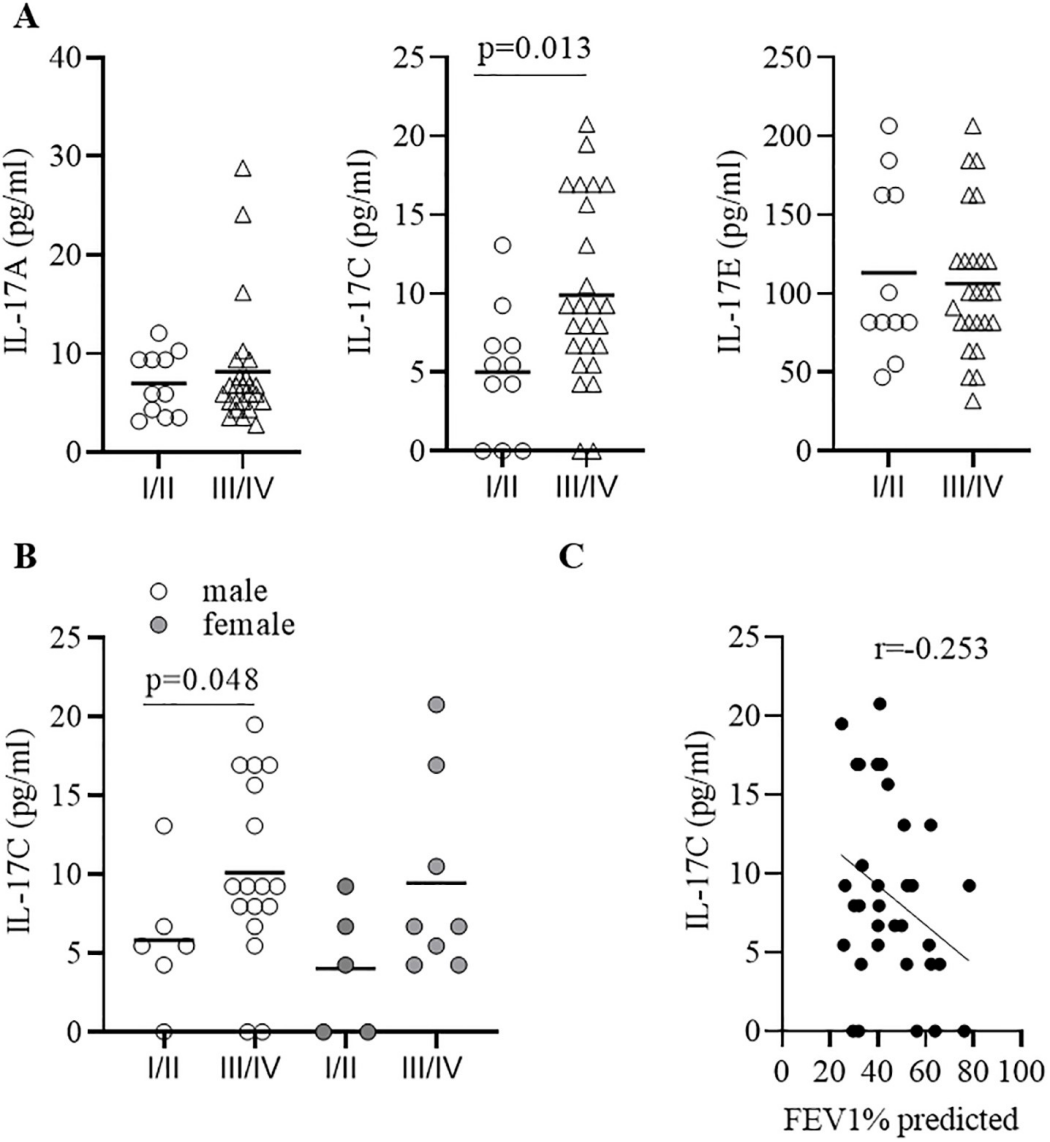
IL-17C concentrations are increased during advanced COPD. (A) Concentrations of IL-17A, IL-17C, and IL-17E in sputum collected from GOLD I/II and GOLD III/IV COPD patients during AECOPD. (B) Concentrations of IL-17C in sputum separated in male (m) and female (f) donors. Horizontal lines indicate mean values. (C) Association of IL-17C concentrations with FEV1 (%) predicted. Correlation analysis was performed using nonparametric Spearman’s correlation test.

**Fig 7:**
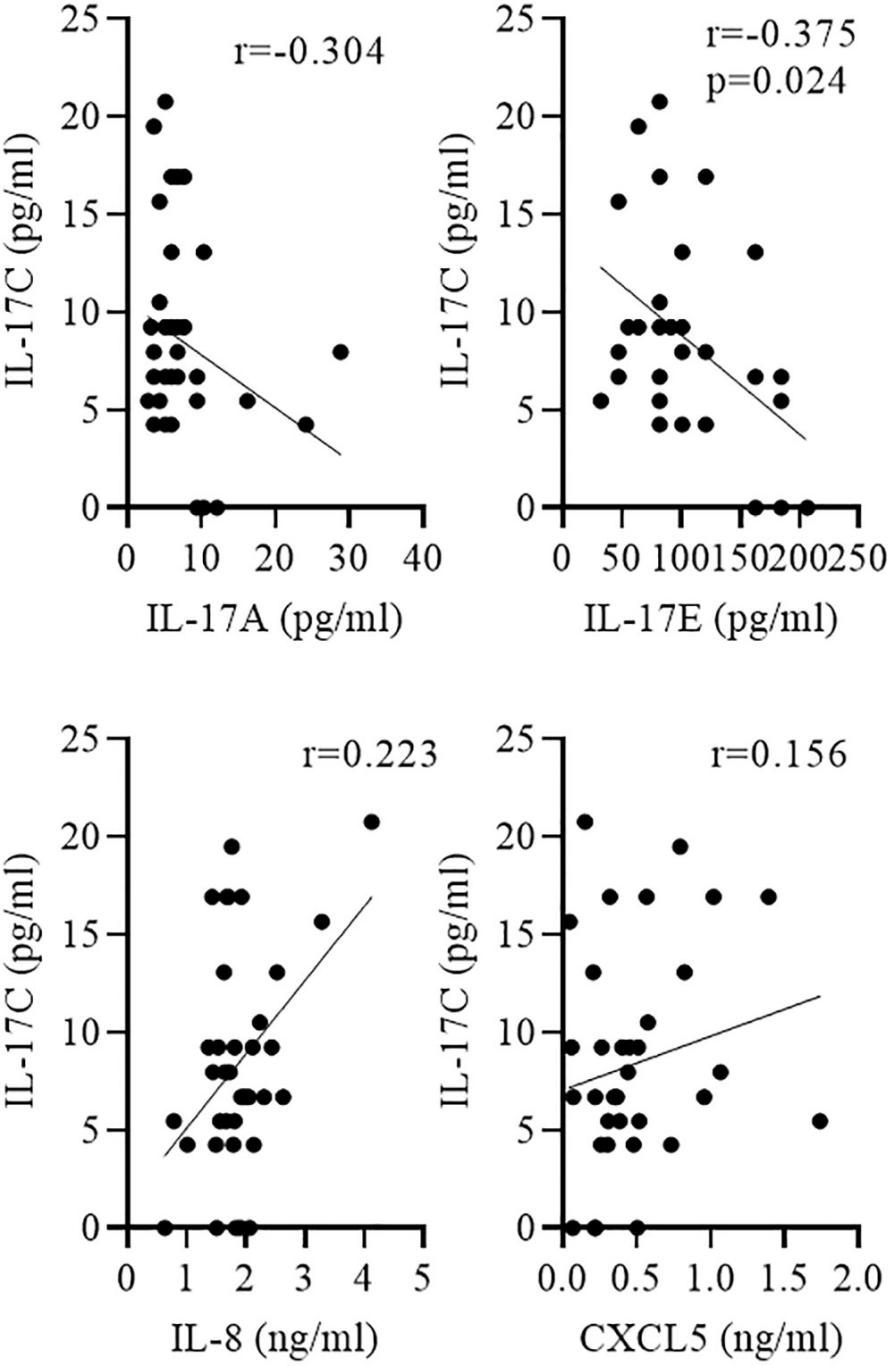
Negative correlation between IL-17C and IL-17E concentrations. Sputum was collected from COPD patients during AECOPD. Correlation of IL-17C with indicated cytokines was tested using nonparametric Spearman’s correlation test.

We measured the concentrations of the IL-17 cytokines IL-17A, IL-17C, and IL-17E and the chemokines IL-8 and CXCL5 in sputum samples from 36 patients collected during AECOPD. The characteristics of the patients are summarized in Table 1. Subgroup analysis showed an association between IL-17C concentrations and disease severity. Concentrations of IL-17C were significantly increased during advanced COPD (GOLD III/IV) compared to moderate (GOLD I/II) COPD, whereas concentrations of IL-17A, IL-17E, IL-8, and CXCL5 did not associate with disease severity (Fig 6A and Table 2). There were no significant differences in the concentrations of IL-17C between males and females (Fig 6B). Concentrations of IL-17C negatively associated with the lung function of the COPD patients (defined as FEV1% predicted, Fig 6C), however without reaching statistical significance. Moreover, the number of pack-years did not correlate with IL-17C concentrations (r = -0,167). IL-17C concentrations correlated negatively with IL-17E concentrations and positively, but not significantly with IL-8 concentration (Fig 7).

**Table 1:** Patient characteristics.

Parameter	GOLD I/II	GOLD III/IV	P value
N	11	25	
Sex (female/male)	5/6	8/17	ns
Age (years)	70.27±8.93	69.2±11.95	ns
Current smokers	3/11	10/15	ns
pack years	19.00±19.27	48.88±35.98	0.014
FEV1 (% predicted)	63.45±9.44	38.17±8.95	<0.0001

ns = not significant, GOLD = Global Initiative for Chronic Obstructive Lung Disease; values are mean±SD.

**Table 2:** Concentrations of cytokines.

Parameter	GOLD I/II	GOLD III/IV	P value
IL-17A (pg/ml)	6.99 (3.19)	8.16 (6.18)	0,9002
IL-17C (pg/ml)	5.01 (4.07)	9.90 (5.75)	**0.013**
IL-17E (pg/ml)	113.31 (55.26)	106.4 (45.26)	>0,9999
IL-8 (ng/ml)	1.61 (0.51)	1.99 (0.64)	0.079
CXCL5 (ng/ml)	0.48 (0.47)	0.49 (0.33)	0.68

GOLD = Global Initiative for Chronic Obstructive Lung Disease; values are mean±SD.

## Discussion

Our study indicates that IL-17C is present in spontaneous sputum of COPD patients collected during AECOPD and that IL-17C levels associate with disease severity. In our pre-clinical models of acute and chronic lung inflammation we show that the deficiency for IL-17C results in a reduced recruitment of neutrophils into the lung and decreased destruction of lung tissue.

Neutrophilia is central in the pathogenesis of COPD [25]. Neutrophils drive the progression of COPD through multiple mechanisms, such as the release of proteases, exosomes, and reactive oxygen species [25, 26]. Thus, it is of interest to identify inflammatory mediators that promote neutrophilic inflammation in stable disease and during AECOPD and therefore qualify as therapeutic targets. As lungs of stable COPD patients are frequently infected with bacteria and infections triggered by bacteria and viruses associate with AECOPD it is obvious that immune responses required for the elimination of microbes are activated in lungs of COPD patients [27]. Studies by Roos et al. showed, for instance, that IL-17A and IL-8 are elevated in sputum during NTHi-associated AECOPD and that IL-17A is present in end-stage COPD [28, 29]. IL-17A and IL-8 mediate the elimination of extracellular pathogens through the recruitment of neutrophils [30]. In this study, we measured the concentrations of IL-17C, a cytokine also known to mediate the recruitment of neutrophils [4, 11, 13, 19, 31], in spontaneous sputum during AECOPD. We show for the first time, to our knowledge, that IL-17C is present in sputum of COPD patients and provide evidence that concentrations of IL-17C associate with disease severity (GOLD I/II vs. GOLDIII/IV). We further found a negative association of IL-17C levels with the lung function (FEV1% predicted), which, however, did not reach statistical significance. We also measured the concentrations of IL-17A and IL-17E and the concentrations of the neutrophilic chemokines IL-8 and CXCL5. We detected IL-17A and IL-8 at comparable concentrations as Roos et al. [28]. However, we did not find a significant association of these cytokines and chemokines with disease severity.

Our patients study has limitations and is preliminary. We did not analyze concentrations of IL-17C at stable state and in healthy controls. Moreover, we were not able to evaluate the cause of the exacerbation and the presence of bacterial or viral infection. Therefore, our data do not indicate whether or not IL-17C concentrations associate with specific viral or bacterial infections. To clarify whether IL-17C levels correlate with certain respiratory pathogens, such as NTHi, as well as with the expression of neutrophilic chemokines and lung function our findings require verification in a second and significantly larger cohort. Future longitudinal studies need to show whether IL-17C is correlated with severity and progression of the disease and, thus, therefore can serve as a biomarker in COPD.

We have shown before that sub-chronic and chronic NTHi-induced inflammation promotes the recruitment of neutrophils in lung cancer models in an IL-17C-dependend manner [13, 32]. In line with our previous work, we found that numbers of pulmonary neutrophils were significantly reduced in *Il-17c*^*-/*-^ mice after the exposure to the combination of CS and NTHi. Thus, IL-17C possible contributes to pulmonary inflammation during AECOPD triggered by bacteria, such as NTHi. To explore a possible function of IL-17C in tissue destruction associated with chronic neutrophilic inflammation, we exposed WT and *Il-17c*^*-/*-^ mice to NTHi for up to 12 weeks and determined the inflammatory score and MCL. Exposure to NTHi resulted in neutrophilia and tissue destruction which was attenuated in *Il-17c*^*-/*-^ mice. This finding further supports the hypothesis that IL-17C promotes the progression of COPD through the recruitment of neutrophils into the lung. As outlined in the introduction, Jamieson et al. demonstrated, in a recent study, that the release of IL-17C from cultured airway epithelial cells is synergistically induced by pathogens known being involved in AECOPD (e.g. rhinovirus and Gram-negative bacteria) and that its expression is increased in cells obtained from COPD patients [11]. Moreover, we could detect IL-17C in human bronchial tissue by immunohistochemistry in paraffin-embedded human bronchial samples from COPD patients [5]. Tissue culture studies also demonstrated that knockdown of IL-17C or its receptor IL-17RE in airway epithelial cells reduces the expression of inflammatory mediators including neutrophilic chemokines in response to infection [5, 8, 11]. In addition, knockdown of IL-17C decreased neutrophil chemotaxis *in vitro* [11]. Together, these pre-clinical studies point to a function of IL-17C in acute and chronic neutrophilic lung inflammation triggered by COPD pathogens.

Our pre-clinical study has limitations. We did not test whether the deficiency for IL-17C affects myeloid differentiation of hematopoietic stem cells. Therefore, it is possible that IL-17C not only mediates neutrophilic inflammation in the lung locally via the induction of cytokines and chemokines, but also through differentiation of hematopoietic stem cells. In our acute and chronic models, numbers of pulmonary neutrophils and concentrations of G-CSF were decreased in mice deficient for IL-17C after exposure to inactivated NTHi. However, to test whether IL-17C mediates the recruitment of neutrophils in a model of NTHi-induced AECOPD, mice should be chronically exposed to CS and infected with viable NTHi. Moreover, in our study, we only included female mice. As hormone (e.g. estrogen) levels can influence immune responses, sex-specific effects cannot be excluded in our experiments.

It also should be noted that IL-17C has a function in prevention and elimination of infections [3, 4, 33]. IL-17C is involved in the expression of antimicrobial peptides and the IL-17C mediated recruitment of neutrophils likely has a function in the elimination of bacteria at mucosal surfaces [8, 9, 4]. Moreover, IL-17C is involved in the recruitment and regulation of lymphocytes. IL-17C has, for instance, been shown to regulate Th17 cells [34–36]. IL-17A expressing immune cells, such as Th17 cells, are involved in the elimination of extracellular lung pathogens, such as *Streptococcus pneumoniae* and *Klebsiella pneumonia* [37]. Therefore, IL-17C has a potentially protective activity at epithelial surfaces and its neutralization may result in an increased risk for lung infections. Future studies need to investigate the function of IL-17C during acute and chronic infection with viable NTHi.

In summary, our study provides additional insight into the contribution of IL-17C in the development of COPD. We demonstrate that IL-17C is present in sputum during AECOPD and provide evidence that IL-17C associates with disease severity. We show that IL-17C promotes NTHI-induced neutrophilia in a mouse model of acute and chronic COPD-like inflammation. The results presented here together with the above discussed studies suggest IL-17C as a therapeutic target in COPD, especially during AECOPD. Therapeutic anti-IL-17C-antibodies which are currently developed for the treatment of atopic dermatitis [38] could be implemented in clinical studies.

## Supporting information

S1 Fig IL-17C mediates chronic neutrophilic inflammation. WT and Il-17c-/- mice were exposed three times per week to NTHi at days 1, 3, and 5 within the first 4 weeks and once a week at day 1 in the following 8 weeks. (A) Numbers of total immune cells, neutrophils, macrophages, and lymphocytes were determined in BAL fluids 24 hours after the final exposure to NTHi (n = 3–4 per group). (B) Representative lung histology hematoxylin and eosin staining) and inflammatory score (n = 4 per group, scale bar: 200 μm). Data were compared by unpaired Student’s t-test and are shown as the mean ± SD. *p < 0.05 and **p < 0.01.
(PDF)
