# Supplementary material for: IL-17C contributes to NTHi-induced inflammation and lung damage in experimental COPD and is present in sputum during acute exacerbations
Source: PLoS One. 2021 Jan 7;16(1):e0243484. doi: 10.1371/journal.pone.0243484 (PMC7790230; doi:10.1371/journal.pone.0243484)
Supplement: S1 Fig — WT and Il-17c-/- mice were exposed three times per week to NTHi at days 1, 3, and 5 within the first 4 weeks and once a week at day 1 in the following 8 weeks. (A) Numbers of total immune cells, neutrophils, macrophages, and lymphocytes were determined in BAL fluids 24 hours after the final exposure to NTHi (n = 3–4 per group). (B) Representative lung histology hematoxylin and eosin staining) and inflammatory score (n = 4 per group, scale bar: 200 μm). Data were compared by unpaired Student’s t-test and are shown as the mean ± SD. *p < 0.05 and **p < 0.01. (PDF) [file pone.0243484.s001.pdf]

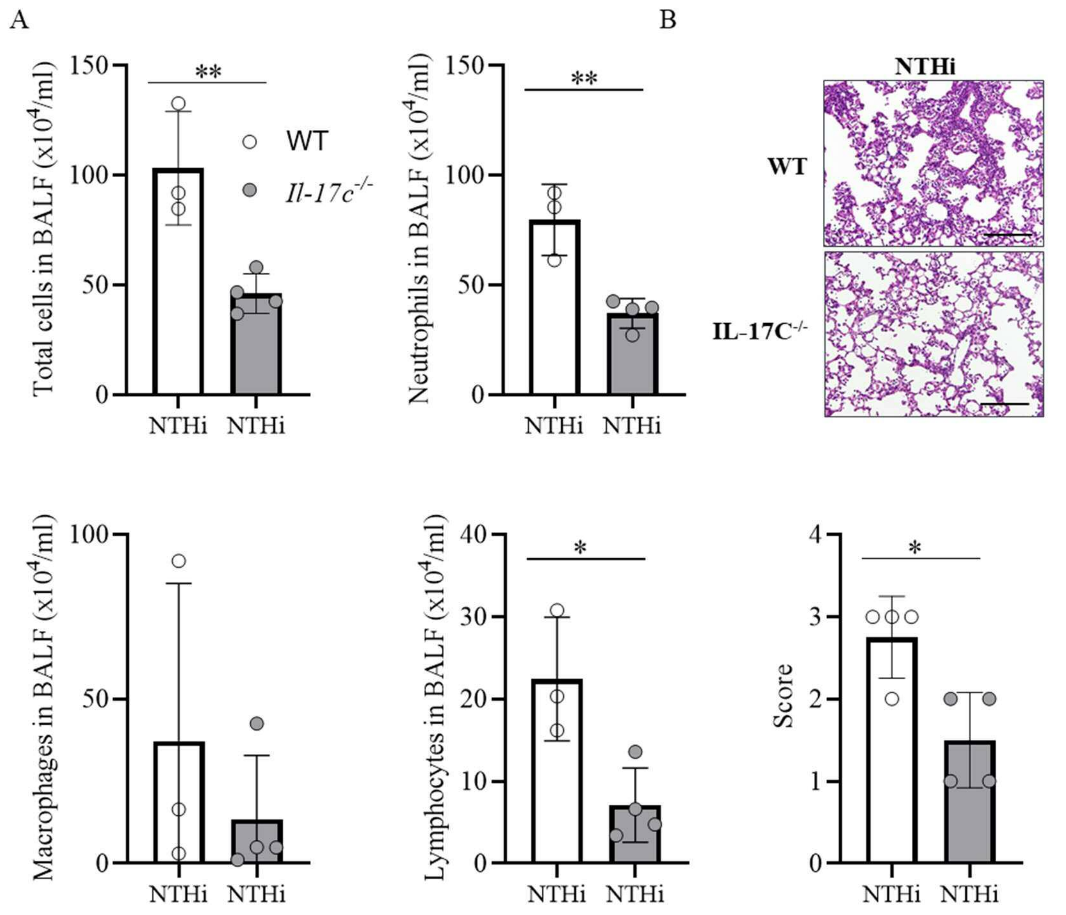

S1 Fig. IL-17C mediates chronic neutrophilic inflammation. WT and *Il-17c*$^{-/-}$ mice were exposed three times per week to NTHi at days 1, 3, and 5 within the first 4 weeks and once a week at day 1 in the following 8 weeks. (A) Numbers of total immune cells, neutrophils, macrophages, and lymphocytes were determined in BAL fluids 24 hours after the final exposure to NTHi (n=3-4 per group). (B) Representative lung histology hematoxylin and eosin staining) and inflammatory score (n=4 per group, scale bar: 200 μm). Data were compared by unpaired Student's t-test and are shown as the mean ± SD. $^*p < 0.05$ and $^{**}p < 0.01$.
